# Beyond the blockade: unmet needs in systemic targeted atopic dermatitis therapy

**DOI:** 10.3389/fimmu.2025.1712757

**Published:** 2025-11-27

**Authors:** Li Zhang, Ge Peng, Mingyue Wang, François Niyonsaba, Xinghua Gao

**Affiliations:** 1 Department of Dermatology, The First Hospital of China Medical University, Liaoning, China; 2 Key Laboratory of Immunodermatology, Ministry of Education and National Health Commission, National Joint Engineering Research Center for Theranostics of Immunological Skin Diseases, Liaoning, China; 3 Atopy (Allergy) Research Center, Juntendo University Graduate School of Medicine, Tokyo, Japan; 4 Faculty of International Liberal Arts, Juntendo University, Tokyo, Japan

**Keywords:** atopic dermatitis, targeted therapies, biologics, JAK inhibitors, blockade

## Abstract

Current atopic dermatitis treatments have been revolutionized by systemic targeted therapies that modulate inflammatory cytokine signaling pathways. While agents such as Janus kinase inhibitors and interleukin-4/interleukin-13 pathway inhibitors have shown significant efficacy, unmet needs persist. These needs include challenges in achieving stable disease control and remission, addressing nonresponders, managing potential side effects, and alleviating the ongoing struggle with pruritus. Future directions will focus on developing dual/multitarget drugs, creating longer-acting formulations, improving administration convenience, reducing dosing frequency, identifying novel therapeutic targets, and incorporating patient-reported outcomes in clinical assessments.

## 1 Introduction

Atopic dermatitis (AD) is a prevalent chronic inflammatory skin condition, which are not associated with autoimmune diseases but are instead linked to IgE-mediated conditions such as asthma, rhinitis, conjunctivitis, and food allergy (1). In the United Kingdom alone, approximately one in five children and one in ten adults are affected by AD (2), whereas in developed countries overall, the prevalence of AD is estimated to range from 15 to 20% (3). Clinically, AD is characterized by dry, intensely itchy skin, often accompanied by redness (erythema) and thickening of the skin (lichenification), which significantly impair patients’ quality of life. Relentless itch and visible skin changes often result in sleep disturbances, anxiety, depression, social isolation, and reduced productivity, highlighting the broader psychosocial toll of the disease (4, 5).

**Figure 1:**
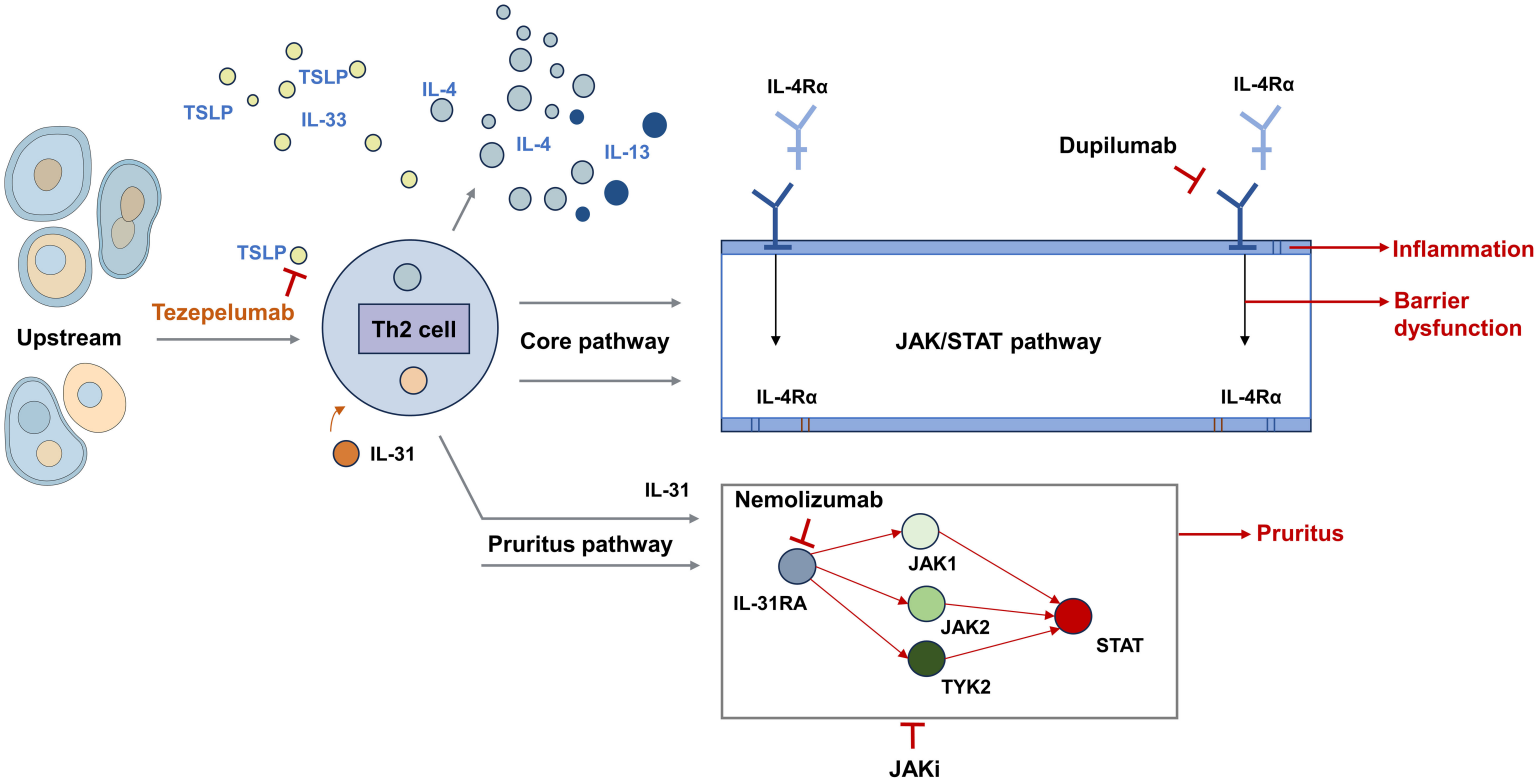
Key pathogenic pathways and therapeutic targets in AD.

The intricate pathophysiology of AD involves a complex interplay of genetic predisposition, immune dysregulation, skin barrier dysfunction, and environmental factors (5–8). A dysregulated T helper 2 (Th2) immune response plays a central role, triggering the overproduction of type 2 cytokines such as interleukin (IL)-4, IL-5, IL-13, IL-31 and thymic stromal lymphopoietin (TSLP), as well as histamine release and eosinophilic infiltration (9). These cytokines not only drive characteristic pruritus and inflammation but also perpetuate epidermal barrier impairment. In chronic AD, immune polarization often shifts toward the Th1 and Th17 pathways, contributing to sustained inflammation and epidermal hyperplasia (7). Notably, many of the abovementioned cytokines exert their biological effects through the Janus kinase/signal transducer and activator of transcription (JAK/STAT) signaling cascade, which plays a pivotal role in amplifying and sustaining the inflammatory milieu in patients with AD (10, 11). The identification of these specific immune pathways and their driving cytokines has been pivotal for understanding AD pathogenesis, paving the way for the development of targeted therapeutic agents (**Figure 1**).

Recent years have witnessed a paradigm shift in AD treatment, which has transitioned from nonspecific immunosuppressive to targeted therapies, including both biologic agents and small-molecule inhibitors. These therapies include both biologic agents targeting extracellular cytokines and their receptors, as well as small-molecule inhibitors, particularly JAK inhibitors, that act intracellularly to block multiple cytokine signals simultaneously, providing more effective and safer treatment options for patients with moderate-to-severe disease who do not respond adequately to conventional topical therapies or for whom such treatments are contraindicated (12, 13). While the development of novel topical agents is also a rapidly advancing field, this review will focus primarily on the progress, unmet needs, and future directions of systemic targeted therapies for patients with moderate-to-severe AD.

## 2 Current targeted therapies for AD: an overview

The current therapeutic landscape of AD has undergone a major transformation with the advent of targeted therapies designed to modulate key immune pathways involved in disease pathogenesis. These therapies primarily include small-molecule JAK inhibitors and monoclonal antibodies targeting specific ILs and their receptors (**Table 1**) (12, 14).

**Table 1:** Approved targeted therapies for AD.

Class	Drug name	Target/mechanism	Administration	Key indication (moderate-to-severe AD)	Key efficacy	Key adverse effects
JAK Inhibitors	Abrocitinib	JAK1	Oral	≥12 years	Rapid onset. EASI-75 (wk 12): ~70.3%. Superior to dupilumab in head-to-head trial.	Nausea, headache, herpes simplex. Black box warning.
	Upadacitinib	JAK1	Oral	≥12 years	Very rapid onset. EASI-75 (wk 16): ~70-80%. Superior efficacy to dupilumab.	Acne (prominent), elevated CPK. Black box warning.
	Baricitinib	JAK1/JAK2	Oral	Adults (Europe ≥2 years)	Rapid onset. EASI-75 (wk 16): ~30-40%.	Box warning
	Ruxolitinib	JAK1/JAK2	Topical	Mild-to-moderate AD (≥2 years)	Significant EASI/IGA improvement vs. vehicle.	Application site reactions.
Biologics	Dupilumab	IL-4Rα (blocks IL-4 & IL-13)	Subcutaneous	≥6 months	Strong long-term safety. EASI-75 (wk 16): ~40-60%. 39% IGA 0/1 at Wk 52. Slower onset.	Conjunctivitis (up to 20%), injection site reactions
	Tralokinumab	IL-13	Subcutaneous	≥12 years	EASI-75 (wk 16): ~30% (adults), 21.4% (adolescents). Slower onset.	URTI, conjunctivitis (potentially lower risk than dupilumab).
	Lebrikizumab	IL-13	Subcutaneous	≥12 years	EASI-75 (wk 16): ~40%. Slower onset.	Injection site reactions, conjunctivitis.
	Nemolizumab	IL-31Rα	Subcutaneous	Severe AD w/pruritus (≥13 years, Japan)	Significant & rapid pruritus reduction. Modest EASI improvement.	Eczema flares, eczemoid dermatitis, peripheral edema, staphylococcal skin infection.

### 2.1 JAK inhibitors

JAK inhibitors are a class of small-molecule drugs that have shown significant efficacy in the treatment of moderate-to-severe AD (12). They function by inhibiting the JAK/STAT signaling pathway, which is essential for the signaling of most ILs involved in the progression of AD (15). Four JAK family members, namely, JAK1, JAK2, JAK3, and tyrosine kinase 2 (TYK2), have been identified and form homodimers and heterodimers to mediate cytokine signaling. By blocking the activity of these kinases, JAK inhibitors can simultaneously interfere with the signaling of multiple proinflammatory cytokines, including IL-4, IL-13, IL-31, IL-17, IL-22, and interferon-gamma, which are involved in AD (16). This broad inhibition helps to modulate the Th2 cell- and Th17-mediated inflammation, epidermal barrier dysfunction, and itch signaling that are characteristics of AD.

The clinical application of JAK inhibitors has revolutionized the treatment of AD by offering effective systemic and topical options. Oral selective JAK1 inhibitors, such as abrocitinib and upadacitinib, provide rapid and profound symptom relief (17). In the JADE DARE head-to-head trial, abrocitinib (200 mg) was superior to dupilumab in achieving a 75% improvement in Eczema Area and Severity Index (EASI-75) response (70.3% *vs* 58.1%) and itch relief at week 12 (18). Similarly, upadacitinib (30 mg) has shown trends toward superior efficacy compared to dupilumab (12). Furthermore baricitinib, a JAK1/JAK2 inhibitor, has been approved in Europe for use in pediatric patients aged two years and older (19). Additionally, ruxolitinib is approved for patients aged 12 years and older for oral formulations used in the treatment of graft-versus-host disease and for topical use in vitiligo. Recently, the topical formulation (Opzelura) has been approved by the FDA for use in pediatric patients aged 2 years and older for AD, with the caveat that its safety and efficacy have not been established in children under 2 years of age (20, 21). Clinical trials have consistently demonstrated that both oral and topical JAK inhibitors can rapidly ease itching, reduce inflammation, and improve EASI scores (22).

Despite their high efficacy, the primary challenge of JAK inhibitors lies in their safety profile. Common adverse events include nausea, headache (more common with abrocitinib), acne (prominent with upadacitinib), and elevated creatine phosphokinase (CPK) levels (17). More significant concerns relate to the “black box” warnings issued by regulatory agencies for increased risks of serious infections (including herpes zoster), malignancy, major adverse cardiovascular events, and venous thromboembolism (22, 23). However, it is important to contextualize this risk: studies have suggested that the elevated risk for serious adverse events (including major adverse cardiovascular events, malignancy, and venous thromboembolism) is predominantly observed in patients with pre-existing risk factors, such as older age and a history of cardiovascular disease (24). Therefore, the potential for adverse events necessitates a careful consideration of the risk–benefit profile of each patient before initiating treatment with a JAK inhibitor.

### 2.2 IL-4 and IL-13 pathway inhibitors

IL-4 and IL-13 are key drivers of type 2 inflammation in AD. IL-4 is considered an orchestrator of Th2 cell polarization and immunoglobulin (Ig) E class switching, whereas IL-13 plays a major role in driving inflammation at the tissue level, particularly in the skin. Several targeted therapies have been developed to inhibit signaling by these cytokines, offering effective treatments for moderate-to-severe AD (16).

Dupilumab and stapokibart are two fully human monoclonal antibodies that target IL-4 receptor (IL-4R), thereby blocking the signaling of both IL-4 and IL-13 (25). In contrast, tralokinumab and lebrikizumab selectively inhibit IL-13 signaling, and both drugs have been approved for use in adolescents and adults aged 12 years and above with AD (26, 27). These inhibitors of the IL-4 and/or IL-13 pathways have shown significant efficacy in achieving clearer skin, improving skin barrier function, and reducing the intense itching associated with AD. Their safety profiles are generally favorable, with common adverse reactions being injection site reactions. However, a notable limitation associated specifically with IL-4Rα blockade (dupilumab) is a significantly higher incidence of conjunctivitis, which can affect up to 20% of patients (28). While generally mild to moderate, this conjunctivitis requires monitoring and ophthalmic management (25). Tralokinumab and lebrikizumab also carry a risk of conjunctivitis, though some data suggest it may be less frequent than with dupilumab (26, 29).

As the first biologic agent approved for AD, dupilumab has significantly altered the treatment paradigm for patients whose disease is not adequately controlled with topical therapies (30, 31). By blocking IL-4R, dupilumab effectively inhibits the signaling pathways of both IL-4 and IL-13, two key cytokines that drive type 2 inflammation in AD. This dual blockade leads to a downregulation of type 2 inflammatory gene expression, a reduction in epidermal hyperplasia, and the restoration of skin barrier function (32). Importantly, dupilumab does not act as classical immunosuppressants, thereby avoiding the broad effects on the immune system typically associated with traditional systemic therapies. Extensive clinical trial data have consistently demonstrated the significant efficacy of dupilumab in treating moderate-to-severe AD (33, 34). For example, in the foundational LIBERTY AD CHRONOS trial, 39% of adults receiving dupilumab (300 mg) with topical corticosteroids (TCS) achieved IGA 0/1 (clear or almost clear skin) at week 52, compared to 12% in the placebo + TCS group (28). Importantly, long-term studies have shown that the efficacy and safety of dupilumab can be sustained for up to five years with continuous treatment (30, 31). The newer IL-13 inhibitors also show robust efficacy; tralokinumab, in the ECZTRA 6 trial for adolescents, demonstrated an EASI-75 response of 21.4% (*vs* 4.3% placebo) at week 16 (29), and lebrikizumab has shown similar results (26). Additionally, treatment with dupilumab has been associated with reductions in the frequency of AD exacerbations and the need for hospitalization, further supporting its role in long-term disease control. Dupilumab has been approved for the treatment of patients with moderate-to-severe AD for more than six months in multiple countries (13). Compared with JAK inhibitors, anti-IL-4Rα therapies may have a slower onset of action but are generally preferred for patients who require a well-characterized long-term safety profile.

### 2.3 IL-31 receptor antagonists

IL-31 is a cytokine that has been identified as a key player in the induction of pruritus, the hallmark symptom of AD (35). Elevated levels of IL-31 are correlated with the severity of AD and the intensity of itching. Nemolizumab is a humanized monoclonal antibody that acts as an antagonist of the IL-31 receptor, specifically blocking the IL-31 receptor (IL-31R), which forms a heterodimeric complex with the oncostatin M receptor. By targeting IL-31, nemolizumab helps disrupt the itch–scratch cycle that significantly contributes to the exacerbation of AD (36). Apart from its approval in Japan, nemolizumab has now also been approved for the treatment of moderate-to-severe AD in the United Kingdom, the European Union, Switzerland, and the United States (36–40). Clinical trials have demonstrated its efficacy in reducing pruritus and improving overall disease severity, as measured by EASI scores. Reported adverse events associated with nemolizumab include eczema flares, eczemoid dermatitis, peripheral edema, and staphylococcal skin infection, but overall tolerability remains acceptable in most patients (36).

### 2.4 TSLP inhibitors

TSLP is an epithelial cell-derived cytokine that acts as an upstream initiator of type 2 immune responses by activating dendritic cells, mast cells and Th2 lymphocytes. Moreover, high TSLP expression has been observed in both the skin lesions and serum of patients with AD, indicating that TSLP is a promising therapeutic target (6, 41). Tezepelumab, a human IgG2 monoclonal antibody, inhibits circulating TSLP by blocking its interaction with the TSLP receptor, thus suppressing downstream inflammatory cascades (42). While tezepelumab has shown some efficacy in achieving an EASI-50 response in a phase 2a trial, it did not reach statistical significance for other key disease endpoints (43). Additionally, small-molecule inhibitors of the TSLP receptor are under development as potential topical therapeutic options for AD.

## 3 Comparative analysis of targeted therapies for AD

With the increasing number of targeted therapies available for AD, comparing their efficacy and safety profiles is crucial to guide treatment decisions and identify optimal strategies for patients with different profiles.

### 3.1 Contrasting efficacy and safety profiles of biologics and JAK inhibitors

Network meta-analyses have been conducted to compare the efficacy of different biologics and JAK inhibitors in the treatment of AD. These analyses suggest that compared with other therapies, upadacitinib, an oral JAK inhibitor, is often noted for having the fastest onset of action, providing rapid symptom relief, and may present the most favorable response estimates for achieving high levels of skin clearance (EASI-90, EASI-75, and IGA 0/1), but it may be associated with higher rates of adverse events (44). Tralokinumab, an IL-13 inhibitor, may be particularly effective at alleviating pruritus (40), and it is important to highlight nemolizumab, which targets the IL-31 receptor, for its strong and specific efficacy in reducing pruritus, the hallmark symptom of AD. Dupilumab, which targets both IL-4 and IL-13, consistently has better outcomes than the placebo does across various measures of disease severity in pediatric patients (13, 30).

Real-world evidence comparing oral JAK inhibitors and dupilumab in adult patients suggests that while oral JAK inhibitors do not appear to increase the risks of major adverse cardiovascular events, venous thromboembolism, renal events, or malignancies in AD patients, they may be associated with higher risks of skin and subcutaneous tissue infections, herpes infections, and acne than dupilumab (45). Conversely, the use of dupilumab may increase the risk of ophthalmic complications. These comparative data highlight a landscape of crucial therapeutic trade-offs. The “optimal” drug varies depending on the specific outcome prioritized. For example, regarding efficacy, some network meta-analyses suggest oral JAK inhibitors like upadacitinib may provide the highest rates of skin clearance (e.g., EASI-90) (44), whereas other biologics like tralokinumab may be particularly effective for the specific outcome of pruritus relief (40). This variation extends to safety profiles, where a patient and clinician must weigh distinct risks: real-world evidence suggests oral JAK inhibitors may carry higher risks of certain infections (like herpes infections or acne) (45), which must be balanced against the increased risk of ophthalmic complications, such as conjunctivitis, associated with dupilumab (45). Therefore, these findings indicate that while dupilumab remains a highly effective and well-established treatment option (46), the selection of newer JAK inhibitors and other biologics must be tailored to different patient profiles based on their specific symptoms (e.g., predominant itch *vs*. skin lesions) and tolerance for different safety considerations (46).

### 3.2 Identifying optimal treatment strategies based on patient profiles

Clinical guidelines for AD management such as the AAD and EuroGuiDerm guidelines strongly recommend biologics and JAK inhibitors as first-line systemic options for moderate-to-severe AD when standard topical therapies have failed (47–49). However, the selection of the most appropriate targeted therapy must be based on a comprehensive assessment of the individual patient’s profile, including efficacy expectations, speed of onset, safety considerations, comorbidities, age, and personal preferences (50). For instance, in patients with severe, highly symptomatic disease requiring rapid control of skin lesions and intense pruritus, oral JAK inhibitors (especially high doses) may be advantageous. A head-to-head clinical trial (Heads Up) demonstrated that upadacitinib (30 mg) was superior to dupilumab for achieving EASI-75 at week 16 (71.0% *vs* 61.1%) (51), and showed significant advantages in speed of onset, with superior itch improvement as early as week 1 (51) Network meta-analyses also support that high-dose upadacitinib and abrocitinib demonstrate the highest relative efficacy (50, 52). This efficacy advantage, however, must be balanced against their safety profile. JAK inhibitors carry “black box” warnings (21), and have shown higher rates of serious infections, eczema herpeticum, and herpes zoster in clinical trials (51, 53). Therefore, for patients with a history of recurrent herpes infections, high risk of serious infection, cardiovascular risk factors, or a history of malignancy, a biologic with a well-established long-term safety profile (30, 31), such as dupilumab, is often the preferred first-line targeted therapy.

Comorbidities are another critical factor influencing selection. For example, the most common adverse event associated with dupilumab is conjunctivitis (51, 54); thus, for patients with pre-existing severe ocular disease, clinicians may prefer other IL-13 specific inhibitors (e.g., tralokinumab or lebrikizumab) (26, 27) or a JAK inhibitor. Age and patient preferences are also key determinants. Dupilumab is approved for infants (≥6 months) (13), whereas baricitinib is approved for children (≥2 years) in Europe (19), and specific guideline recommendations must be consulted for special populations such as pregnant or breastfeeding women (47). Furthermore, the convenience of oral JAK inhibitors (45, 55) compared to the subcutaneous injections required for biologics (56, 57) is an important factor in shared decision-making. Additionally, real-world data show that a substantial disease burden remains even for patients on the most widely used systemic therapies like dupilumab (58). For these patients with an inadequate response, the primary strategy is often switching treatment [e.g., to a JAK inhibitor (51)]. If the response remains insufficient after switching, combination therapy (59) may then be considered as a subsequent strategy for more refractory cases. Ultimately, a shared decision-making process between the clinician and the patient is crucial for determining the optimal treatment approach that balances efficacy, safety, and patient preferences.

## 4 Unmet medical needs in the management of AD

Despite the significant advancements in AD treatment with targeted therapies, several unmet medical needs persist, and further research and development are needed to optimize patient outcomes and quality of life.

### 4.1 Pruritus management

Chronic pruritus is a hallmark symptom of AD and is a major contributor to the disease burden and reduced quality of life (60). While targeted therapies often lead to itch reduction, many patients continue to experience persistent or inadequately controlled pruritus. The complex pathophysiology of AD-related itch, involving skin barrier dysfunction, immune dysregulation, and nerve sensitization, makes targeted treatment with single-mechanism therapies challenging. An urgent need exists for highly effective and targeted antipruritic therapies with minimal side effects. Future directions should include mechanistically targeted agents (e.g., neuroimmune modulators) and combination strategies to disrupt the itch–scratch cycle more effectively (16).

### 4.2 Disease control and remission

While current systemic targeted therapies significantly improve symptoms in many patients with moderate-to-severe AD, a substantial proportion (approximately 40–60%) do not achieve adequate disease control (22, 33). Furthermore, 30% of patients fail to reach optimal treatment goals, such as complete or near-complete skin clearance (IGA 0/1) or EASI-90 (61).

A critical distinction must be made between clinical control and true disease remission. Current trial endpoints, such as EASI-75 or IGA 0/1 (51, 62), represent successful disease suppression while the patient is on active therapy. However, a more profound unmet need is the induction of deep and therapy-free remission (TFR)—a state where disease control is maintained long-term even after the treatment— is discontinued (63).

Achieving TFR is exceptionally challenging because AD is a chronic disease with deep-seated immunological and structural persistence. The primary barrier to TFR is the establishment of a “disease memory” within the skin. Groundbreaking research has shown that even after long-term treatment with IL-4Rα blockade (dupilumab) and apparent clinical resolution, pathogenic immune cell populations persist in the resolved skin. These include mature dendritic cells and specialized T helper cells (e.g., TH2A cells) which are absent in healthy controls (64). These lingering cells, along with *Staphylococcus aureus*-specific tissue-resident memory T cells (65), remain primed to reactivate the inflammatory cascade, explaining why disease recurrence upon treatment cessation is so common (64).

This persistent immune memory explains why most current treatments, including biologics and JAK inhibitors, function as highly effective “disease-suppressing” rather than “disease-modifying” agents (63). Patients often require long-term, even lifelong, treatment to maintain control, and symptoms frequently recur upon cessation of therapy (16). Therefore, the development of true disease-modifying therapies that can erase this immune memory, promote immune tolerance, and induce sustained TFR remains one of the most critical unmet needs in AD management (16, 63).

Most current treatments focus on disease suppression rather than the induction of sustained remission. Patients often require long-term, even lifelong treatment, and symptoms frequently recur upon the cessation of therapy. Disease-modifying therapies that not only control symptoms but also alter the natural course of AD, promote immune tolerance, and induce sustained remission or even a functional cure are critically needed (16).

### 4.3 Needs of specific populations

Treatments for AD must be tailored to the unique needs of specific populations. Although dupilumab has extended therapeutic options for children as young as six months, many targeted agents remain unapproved or understudied in pediatric populations, particularly infants and toddlers (13). Moreover, disparities in clinical manifestation, genetic predisposition, and treatment response across racial and ethnic groups are being increasingly recognized. However, most clinical trials underrepresent nonwhite populations, limiting the generalizability of the findings (17). Addressing these gaps requires the intentional inclusion of diverse populations in clinical research and the development of culturally and biologically tailored therapeutic strategies.

### 4.4 Therapeutic inertia

Therapeutic inertia—the delay or failure to escalate treatment when therapeutic goals are not met—is a prevalent obstacle in real-world AD management. Despite the availability of effective targeted therapies, clinicians may hesitate to adjust treatment plans because of concerns about adverse effects, a lack of confidence in newer agents, or the absence of clear escalation protocols (66). This delay in appropriate intervention often results in prolonged patient suffering, poor disease control, and reduced quality of life. Addressing therapeutic inertia will require improved education, updated clinical guidelines, and decision-support tools to facilitate timely treatment optimization.

### 4.5 Cost and accessibility

The high cost of targeted therapies often restricts their accessibility to a broader patient population. Biologic agents and JAK inhibitors remain unaffordable for many patients, particularly in low-resource settings or where insurance coverage is limited. This substantial financial burden may lead to treatment discontinuation or nonadherence, undermining long-term outcomes. Efforts to improve accessibility should include the development of cost-effective therapeutic alternatives, the expansion of biosimilar markets, and policy-level interventions to reduce the economic burden on patients and healthcare systems (55).

### 4.6 Unmet needs in patient-centered care

Despite the increasing clinical understanding of AD pathophysiology and treatment options, patient-centered approaches for AD management remain insufficiently addressed (59). Treatment decisions often prioritize clinical signs and investigator assessments while underemphasizing patient-reported outcomes (PROs), such as itch severity, sleep disruption, fatigue, and emotional distress. Integrating PROs into clinical practice and research is essential for aligning therapeutic goals with patients’ lived experiences. Additionally, real-world evidence (RWE) derived from observational data and routine practice complements findings from randomized clinical trials (RCTs), providing insights into treatment effectiveness, safety, and adherence in diverse patient populations (67). We urge researchers to prioritize the collection and analysis of PROs as primary or secondary endpoints in clinical trials to better address the needs of AD patients. Furthermore, we advocate for the establishment of AD patient registries to collect longitudinal clinical data, including PROs and RWE, to improve our understanding of the natural course of AD, the long-term effects and safety of different treatments, and patterns of disease recurrence. Drawing inspiration from established registries for diseases such as psoriasis, AD registries could provide valuable data to enhance patient care, guide clinical research, and inform the development of more effective treatment strategies (68).

## 5 Future directions in AD therapeutics

The therapeutic landscape of AD is rapidly evolving, driven by ongoing research focused on addressing unmet needs and further improving treatment outcomes. Several promising future directions are currently being explored (**Table 2**).

**Table 2:** Key investigational targeted therapies for AD in late-stage clinical trials.

Drug name	Target	Mechanism of action	Administration route	Phase of development	Potential advantages/unique features
ICP-332	TYK2	Potent and selective TYK2 inhibitor	Oral	Phase 3	Targets TYK2, a member of the JAK family
APG777	IL-13	Novel, subcutaneous monoclonal antibody with an extended half life	Subcutaneous injection	Phase 2	Longer dosing interval, potentially has better potency than lebrikizumab
Barzolvolimab	KIT	Humanized monoclonal antibody that inhibits KIT activity	Subcutaneous injection	Phase 2	Targets mast cell activation
ATI-2138	ITK/JAK3	Oral covalent inhibitor of ITK and JAK3	Oral	Phase 2	Targets T-cell signaling
Rocatinlimab	OX40	Anti-OX40 human monoclonal antibody	Subcutaneous injection	Phase 3	Impedes pathogenic T cells
Difelikefalin	Kappa opioid receptor	Selective kappa opioid receptor agonist outside the CNS	Oral	Phase 3	Targets pruritus without CNS-related side effects
Etrasimod	S1P receptors Subtypes 1,4, and 5	Selective sphingosine 1-phosphate receptor modulator	Oral	Phase 2	Both systemic and localized effects on immune cells
BMX-010	Unknown	Unknown	Topical ointment	Phase 2	Assessing safety and clinical impacts
Rezpegaldesleukin	IL-2/15 receptor	Activates regulatory T cells (Tregs)	Subcutaneous injection	Phase 2	Modulates the immune response by targeting Tregs

### 5.1 Development of dual-target and multitarget agents

One exciting avenue of research is the development of dual-target and multitarget agents, particularly bispecific antibodies that can simultaneously target multiple pathogenic pathways. For example, GB12–09 is a bispecific antibody targeting both IL-4Rα and IL-31Rα (69), whereas NM26–2198 targets IL-4Rα and IL-31, effectively modulating both type 2 inflammation and pruritus (70). These approaches may increase clinical efficacy by overcoming the limitations of single-cytokine blockade, particularly in patients with heterogeneous inflammatory profiles or partial responses to existing monotherapies. Dual or multicytokine blockade may also reduce the likelihood of inflammatory pathway compensation, potentially achieving more sustained disease control.

### 5.2 Exploring longer-acting formulations for improved patient convenience and adherence

Frequent dosing remains a significant barrier to long-term adherence in patients with chronic diseases such as AD. Accordingly, the development of longer-acting formulations of targeted therapies is a key area of innovation. One such agent, APG777, a novel half-life-extended IL-13 monoclonal antibody that is currently in phase 2 clinical trials for AD, has a prolonged half-life of up to 77 days (71). The development of such longer-acting formulations may significantly improve treatment adherence and patient satisfaction by reducing the injection frequency and minimizing the healthcare burden. These formulations are particularly beneficial in pediatric populations and in patients requiring maintenance therapy over extended periods.

### 5.3 Advancements in convenient administration routes

The availability of convenient administration routes is crucial for patient comfort and adherence. While subcutaneous injections are the primary route for most approved biologic therapies, such as dupilumab, stapokibart, tralokinumab, and lebrikizumab, oral JAK inhibitors, such as abrocitinib, upadacitinib, and baricitinib, offer a more convenient oral route of administration. Ongoing research has focused on exploring oral formulations for other targeted therapies as well. For instance, difelikefalin, an oral kappa-opioid receptor agonist, is in phase 3 clinical trials for the treatment of pruritus in patients with AD (72). Similarly, etrasimod, an oral sphingosine 1-phosphate receptor modulator, is undergoing a phase 2 trial for AD (73). The availability of both subcutaneous and oral options provides greater flexibility in treatment selection, allowing clinicians to tailor the route of administration to individual patient preferences and needs, potentially improving treatment adherence and overall satisfaction.

### 5.4 Identification and validation of novel therapeutic targets

Continuous research efforts are focused on identifying and validating novel therapeutic targets involved in the pathogenesis of AD, including exploring targets beyond the canonical type 2 cytokines. For instance, the OX40/OX40 ligand (OX40L) interaction, filaggrin replacement, and modulation of the skin microbiome are areas of active investigation (74). Several therapies targeting other cytokines and receptors, such as IL-22 (e.g., fezakinumab), IL-33, and the components of the IL-23/IL-17 axis, as well as oncostatin M β, are under investigation (75). The drug development pipeline for AD is robust, with numerous investigational drugs targeting various mechanisms of action currently being studied in different phases of clinical trials. These drugs include TYK2 inhibitors (e.g., ICP-332) (76) and ITK/JAK3 inhibitors (e.g., ATI-2138) (56, 57). Additionally, novel approaches such as rezpegaldesleukin, which aims to activate regulatory T cells to suppress inflammation, are being explored (77). The identification and validation of these novel therapeutic targets hold significant promise for the development of new treatments that can address the limitations of current therapies, potentially leading to more effective and personalized approaches for managing AD.

### 5.5 Advancing precision medicine and personalized therapies

A major paradigm shift anticipated in the future of AD management is the move towards precision medicine, transitioning away from the current largely “trial-and-error” approach to selecting systemic therapies (78, 79). AD is increasingly recognized not as a single entity but as a complex syndrome with significant heterogeneity in clinical presentation and underlying molecular mechanisms, referred to as endotypes (80, 81). This heterogeneity explains why patient responses to specific targeted therapies can vary considerably (78).

The development and validation of reliable biomarkers are crucial for realizing the potential of precision medicine in AD (11, 82). Currently, the selection between therapies like IL-4/IL-13 pathway inhibitors (e.g., dupilumab) and JAK inhibitors often relies on clinical factors and shared decision-making, rather than objective biological predictors (78). Future research is intensively focused on identifying predictive biomarkers that can guide therapeutic choices.

Serum biomarkers: Measuring levels of specific cytokines (e.g., IL-13, IL-22, TSLP), chemokines (e.g., CCL17/TARC), or cellular components like eosinophils in peripheral blood holds promise for stratifying patients (83). For example, higher baseline levels of Th2-related markers might predict a better response to IL-4/IL-13 blockade, whereas elevated Th17/Th22 markers could suggest potential benefit from broader-acting JAK inhibitors (80).

Skin transcriptomics and other omics: Analyzing the gene expression profile (transcriptome) directly from skin biopsies allows for a deeper understanding of the patient’s specific inflammatory signature (84). Distinguishing between a predominantly Th2-driven endotype versus a mixed phenotype involving significant Th1, Th17, or Th22 pathway activation could become key for selecting the most appropriate targeted agent (80, 81). Integration with other omics data (epigenomics, proteomics, metabolomics) may further refine these endotypes (84).

Recognizing distinct AD endotypes is fundamental to this personalized approach. Significant differences have been observed across populations. For instance, the “Asian AD phenotype” often exhibits a stronger Th17/Th22 signature compared to the typically Th2-dominant profile seen in European-American patients (13, 85). Other proposed endotypes differentiate based on IgE levels (extrinsic *vs*. intrinsic AD) or filaggrin mutation status (80, 85). Such distinctions imply that future treatment algorithms may need to be tailored; for example, strongly Th2-skewed patients might benefit most from IL-4/IL-13 pathway inhibitors, while those with mixed inflammatory profiles might respond better to JAK inhibitors or potentially therapies targeting IL-17 or IL-22 (75, 80).

Ultimately, precision medicine aims to transform the AD treatment paradigm from the conventional “step-care” approach (where treatments are escalated based on failure) to a biomarker-guided, personalized strategy (78, 86) By leveraging individual patients’ biological profiles, clinicians could select the most effective first-line targeted therapy, improving outcomes, minimizing exposure to ineffective treatments, and optimizing resource utilization (79, 87).

## 6 Clinical guidelines and expert consensus on AD treatment

Clinical practice guidelines and expert consensus statements play vital roles in providing clinicians with evidence-based recommendations for the management of AD, including the use of targeted therapies.

### 6.1 Current recommendations for the use of targeted therapies

The American Academy of Dermatology (AAD) guidelines strongly recommend the use of dupilumab, tralokinumab, abrocitinib, baricitinib, and upadacitinib for the treatment of AD (33). These guidelines reflect the growing body of evidence supporting the efficacy and safety of these targeted agents. Similarly, European guidelines also incorporate dupilumab and other targeted therapies into their recommendations. Expert consensus statements emphasize the increasing importance of biological therapies in the management of pediatric patients with moderate-to-severe AD. A general consensus exists that systemic therapy, including targeted agents, should be considered for patients with moderate-to-severe AD when optimized topical therapy has proven inadequate for controlling the disease (88). These guidelines and consensus statements provide a valuable framework for clinicians in making informed decisions about when and how to incorporate targeted therapies into their treatment algorithms for AD.

### 6.2 Emerging trends and future research priorities highlighted by experts

Experts in the field advocate for setting higher treatment goals in AD management, such as achieving EASI-90 (90% improvement in skin clearance) and a numeric rating scale (NRS) score of 0 or 1 for itch, indicating near-clear skin and minimal to no itch (34). The need to overcome therapeutic inertia by proactively adjusting treatment strategies when patients are not meeting these optimal targets has also been emphasized. Future research priorities include the continued development of disease-modifying therapies that can potentially alter the long-term course of AD, as well as investigating recurrence rates upon the cessation of current treatments. These emerging trends and research priorities underscore the ongoing commitment to further optimize AD management and improve the long-term outcomes of patients.

## 7 Conclusions

Targeted therapies have ushered in a new era in the treatment of AD, offering significant advancements in efficacy and safety, particularly for patients with moderate-to-severe disease. However, despite these remarkable achievements, critical unmet needs persist. A substantial proportion of patients continue to experience inadequate responses to current treatments or unstable disease control. Moreover, the potential for side effects, although generally less severe than traditional immunosuppressants, remains a concern. An inadequate response to existing therapies, residual pruritus, and treatment-limiting side effects underscore the need for improved therapeutic options. Furthermore, therapeutic options for children, especially infants, and for individuals with mild AD are still relatively limited. The persistent burden of pruritus also highlights the need for more targeted antipruritic therapies.

The future of AD therapeutics holds immense promise. The development of dual and multitarget agents, longer-acting formulations, and convenient administration routes are poised to further increase treatment efficacy and support patient convenience. The ongoing identification and validation of novel therapeutic targets have the potential to address the limitations of current therapies and provide new avenues for intervention. The increasing integration of patient-reported outcomes into clinical assessments is crucial for a more holistic evaluation of treatment success, and the growing emphasis on personalized medicine will allow therapeutic strategies to be tailored individual patient profiles and disease endotypes. As researchers continue to elucidate the complex pathogenesis of AD, the field is on the cusp of further breakthroughs that will undoubtedly improve the lives of individuals living with this chronic and often debilitating condition.
